# Inhibitory collaterals in genetically identified medium spiny neurons in mouse primary corticostriatal cultures

**DOI:** 10.1002/phy2.164

**Published:** 2013-11-24

**Authors:** Rupa R Lalchandani, Stefano Vicini

**Affiliations:** 1 Graduate Program in Physiology and Biophysics, Georgetown UniversityWashington, District of Columbia, 20007; 2 Department of Pharmacology and Physiology, Georgetown UniversityWashington, District of Columbia, 20007

**Keywords:** Dissociated culture, dopamine receptors, inhibitory collaterals, striatum, synaptic physiology

## Abstract

Inhibitory collaterals between striatal medium spiny neuron (MSN) subtypes have been shown to critically influence striatal output. However, the low rate of inhibitory collateral detection between striatal MSNs in conventional ex vivo slice recordings has made the study of these connections challenging. Furthermore, most studies on MSN collaterals have been conducted either blind or in models, in which only one MSN subtype can be distinguished. Here, we describe a dissociated culture system using striatal and cortical neurons harvested from genetically modified mice at postnatal day 0. These mice express tdTomato and enhanced green fluorescent protein (EGFP) downstream of the dopamine D1 and D2 receptor promoters, respectively, allowing for simultaneous distinction between the two major subtypes of MSNs. In vitro, these neurons develop spines, hyperpolarized resting membrane potentials and exhibit up-and-down states, while also maintaining expression of both fluorophores through time. Using paired whole-cell patch-clamp recordings from identified MSNs at 14 days in vitro, we are able to detect a much higher rate of inhibitory functional synapses than what has been previously reported in slice recordings. These collateral synapses release γ-Aminobutyric acid (GABA) and shape the firing patters of other MSNs. Although reduced in vitro models have a number of inherent limitations, the cultures described here provide a unique opportunity to study frequently observed functional collaterals between identifiable MSNs. Additionally, cultured neurons allow for control of the extracellular environment, with the potential to investigate pharmacological regulation of inhibitory MSNs collaterals.

## Introduction

The striatum is the sole input nucleus of the basal ganglia and is responsible for projecting signals that result in proper behavior and motor movement control. Nearly 95% of neurons in the striatum are GABAergic medium spiny neurons (MSNs), which express dopamine D1 or D2 receptors and project directly or indirectly to the substantia nigra (SN), respectively. MSNs project local axon collaterals that extend beyond their dense 250–400 *μ*m dendritic tree (Wilson 1994) and integrate GABAergic input from neighboring MSNs at dendrite necks and shafts (Gerfen 1988).

Individual connections between MSNs are quite weak, resulting in reported connectivity rates ranging generally between 0% (Jaeger and Wilson 1994) and 33% (Taverna et al. 2008) in ex vivo brain slices (Plenz 2003). However, a recent study by Chuhma et al. (2011) using channel rhodopsin activation in striatal MSNs suggests MSN-MSN collaterals occur at a rate of 63%. While this investigation studied convergent MSN activity and not individual MSN-MSN synapses, it suggests that MSN connections, when taken together, are critical in shaping striatal output. In addition, evidence points toward specificity in MSN collateral formation, with differences in synapse frequency and strength dependent upon which MSN subtypes are involved (Taverna et al. 2008).

“Feed-back inhibition” between MSNs is critical in integrating and regulating information and has been shown to be strongly affected with changes in dopamine levels (Taverna et al. 2008; Jáidar et al. 2010). It is therefore of compelling importance to study these collaterals in greater detail. Here, we have established a dissociated primary cell culture model of the mouse striatum that allows for increased detection of MSN collaterals. Previous studies have shown an increased rate of synapse formation in in vitro models compared to conventional ex vivo slice preparations (Czubayko and Plenz 2002). We utilize bacterial artificial chromosome (BAC) transgenic mice that express td-Tomato downstream of the D1 receptor promoter and enhanced green fluorescent protein (EGFP) downstream of the D2 receptor promoter to concurrently distinguish between the two MSN subtypes (Gong et al. 2003). With our method, cultures retained expression of their transgene, develop complex dendritic arbor, and exhibit MSN-like firing properties in vitro. In addition, the rate of synapse formation is nearly double of that reported in earlier studies, allowing for a more extensive study of specific MSN collaterals. This model opens the door for targeted studies of MSN collateral synapses that have previously been elusive.

## Material and Methods

### Animals and tissue collection

Mice with BAC D1-tdTomato were crossed with BAC D2- EGFP mice (GENSAT; Gong et al. 2003; Shuen et al. 2008). At postnatal day 0–1 (p0-1), male and female pups were decapitated in accordance with the guidelines of the American Veterinary Medical Association Panel on Euthanasia and the Georgetown University Animal Care and Use Committee. A sterile buffer solution containing 0.7% NaCl, 0.04% KCl, 0.02% KH_2_PO_4_, 0.3% C_6_H_12_O_6_, 0.2% NaHCO3, and 0.001% Phenol Red (all from Fisher Scientific, Hampton, NH) was prepared. Whole brains were removed into the ice-cold buffer solution with the addition of 0.3% bovine serum albumin (Sigma; St. Louis, MO) and 0.01% MgSO_4_ (Fisher Scientific). Meninges were removed and brains were checked for fluorescence using Dual Fluorescent Protein Flashlight (NIGHTSEA, Bedford, MA). EGFP expression was used to guide the exact dissection of striata from brains expressing both fluorophores. Cortices were dissected from littermates that did not express either fluorophore.

### Primary corticostriatal cultures

Once collected, cortical and striatal tissues were minced finely and centrifuged at 1000 rmp (rotations per minute) for 2 min in separate tubes. The supernatants were removed and the pellets were mixed into the buffer solution containing 0.04% trypsin (Sigma). After 15 min in a 37°C water bath shaking at 180 rpm, an equal volume of buffer solution containing 0.001% DNAase, 0.01% trypsin inhibitor, and 0.01% MgSO_4_ was added to the suspensions and centrifuged at 2000 rpm for 5 min. The supernatants were then aspirated and replaced with buffer solution containing 0.01% DNAase, 0.05% trypsin inhibitor, and 0.04% MgSO_4_. After triturating the pellet with fire-fined Pasteur pipettes, an equal volume of buffer solution containing 0.03% MgSO_4_ and 0.001% CaCl_2_ was added. Cells were centrifuged at 1000 rpm for 7 min. The supernatants were removed and pellets were resuspended in 2 mL of Basal Eagle's Medium (Invitrogen, Carlsbad, CA). Cell concentrations of the single-cell suspensions were determined with a hemocytometer. Cells were combined at a 1:3 ratio of cortical to striatal neurons and diluted to a concentration of 5 × 10^5^ cells/mL in Neurobasal Medium supplemented 0.25% glutamine, 1% penicillin-streptomycin, 2% B27 (all from Invitrogen), 50 ng/mL brain derived neurotrophic factor (BDNF; Alomone, Jerusalem, Israel), and 30 ng/mL glial derived neurotrophic factor (GDNF; Sigma). 1.13 cm^2^ glass coverslips had been coated with poly d-lysine (10 μg/mL, Sigma) and incubated in Basal Eagle's Medium; 150 μL of the corticostriatal cell suspension was plated onto each coverslip and incubated at 37°C in 95% O_2_/5% CO_2_ for 40 min; 100 μL was then removed from each coverslip and 200 μL of supplemented Neurobasal Medium was added. At 2 days in vitro (DIV), 250 μL of supplemented Neurobasal Medium was added to each well. Supplemented Neurobasal media were exchanged twice weekly, although BDNF and GDNF were not included after DIV 2 (Tian et al. 2010).

### Electrophysiology

Cells were transferred to the recording chamber in extracellular recording solution (ES) composed of (in mmol/L): NaCl (145), KCl (5), MgCl_2_ (1), CaCl_2_ (1), 4-(2-hydroxyethyl)-1-piperazineethanesulfonic acid (HEPES; 5), glucose (5), sucrose (15), phenol red (0.25 mg/L), and adjusted to pH 7.4 with NaOH (all from Fisher Scientific, Pittsburg, PA). ES was continuously perfused in the chamber at a rate of 2.5 mL/min. Cells were visualized using a Nikon TE-2000S inverted microscope with a 40× phase contrast objective (Tokyo, Japan) and tdTomato and EGFP-expressing neurons were selected. Borosilicate glass capillaries (Wiretrol II, Drummond, Broomall, PA) were pulled on a two-step vertical pipette puller (PP-83; Narishige, Tokyo, Japan). Recording electrodes with resistances between 3 and 5 MΩ were filled with intracellular solution (IS) containing (in mmol/L): K-gluconate (145)**,** HEPES (10), ATP. Mg (5), GTP^**.**^Na (0.2), and ethylene glycol tetraacetic acid (0.5), adjusted to pH 7.2 with KOH (IS_1_). To better study inhibitory collaterals, 145 mmol/L K-gluconate was replaced with a mixture of 100 mmol/L K-gluconate and 44 mmol/L KCl (IS_2_). Recordings were conducted with a 700B amplifier (Molecular Devices, Sunnyvale, CA) and traces were filtered at 1 kHz with an 8-pole low-pass Bessel filter and digitized at 5 kHz using an IBM-compatible microcomputer equipped with Digidata 1322A data acquisition board and pCLAMP10 software.

Drugs were locally exchanged using the Y-tube method (Murase et al. 1989). Stock solutions of bicuculline methobromide (BMR), tetrodotoxin (TTX), 2,3-dihydroxy-6-nitro-7-sulfamoyl-benzo[f]quinoxaline-2,3-dione (NBQX) (all from Abcam, Cambridge, MA) were prepared in water and diluted in ES to a final concentration of 25 *μ*mol/L, 500 nmol/L, and 5 *μ*mol/L, respectively. 4,5,6,7-tetrahydroisoxazolo[5,4-c]pyridin-3-ol (THIP, Sigma) was also prepared in water and diluted in ES to a final concentration of 1 *μ*mol/L. γ-Aminobutyric acid (GABA) (Sigma) was dissolved in water and diluted in ES to achieve final concentrations of 0.3, 1, and 3 *μ*mol/L. Etomidate (Sigma) was prepared in a stock solution with dimethyl sulfoxide (DMSO) and diluted in ES to 5 *μ*mol/L (final DMSO concentration <0.01%).

Recordings from identified neurons were conducted in voltage clamp at −70 mV and current clamp at the cell's resting membrane potential (RMP). Recordings of cells which had leak currents of greater than 100 pA or RMP of less than −50 mV were discarded. Paired recordings were conducted to study synaptic connections between identified MSNs. Two cells were selected and whole-cell patch-clamp configuration was achieved. While in voltage clamp, cells were stimulated (+100 mV, 4 msec) at 3-sec intervals to induce an action potential, and evoked inhibitory postsynaptic currents (eIPSCs) were studied. To monitor series and input resistance, a test voltage pulse was given (−5 mV, 20 msec). Cells with large changes (>20%) in series or input resistance, or unstable holding currents were discarded.

Miniature IPSCs (mIPSCs) were isolated with the presence of TTX and NBQX, and events were detected using semiautomated threshold-based software (Mini Analysis, Synaptosoft, Fort Lee, NJ). At least 100 mIPSC events were averaged per cell and decay phase (*τ*_w_) was determined using a single exponential equation. eIPSC parameters were analyzed in Clampfit 10.2 (Molecular Devices) and averages were based on greater than 20 events. The decay phase of the averaged eIPSC was fit to a double exponential equation.

### Histochemistry

BAC *drd1a*-tdTomato;BAC *drd2*-EGFP mice aged p1, p5, and p10 were killed by decapitation. Whole brains were dissected out and placed into a 4% paraformaldehyde/4% sucrose phosphate buffered saline (PBS) solution at room temperature for 24 h. Brains were then washed 3× with PBS and 150-μm sagittal sections were cut with a Lancer Vibrotome (Series 1000; Sherwood Medical, St. Louis, MO). Slices containing both striatum and substantia nigra were mounted on glass coverslips with VectaShield and visualized with and upright E600FN Nikon microscope.

To better study MSN morphology in vitro, we fixed cultured coverslips with 4% paraformaldehyde/4% sucrose/PBS solution for 15 min, and then rinsed with PBS for 10 min 3×. Cells were permeabilized and blocked as previously reported for striatal cultures (Tian et al. 2010), and incubated with rabbit *α*-GFP (1:200, Invitrogen) for 120 min. After washing, coverslips were transferred into secondary antibody *α*-rabbit Alexa-488 (1:1000) for 60 min and washed 3×. Coverslips were then mounted on slides with VectaShield and visualized. All histochemistry was conducted at room temperature.

### Statistics

Statistical tests and plot graphing were conducted in Prism 5 software (GraphPad) and are further detailed in the *Results* and *Figure Legends*. All data sets were checked for normality using Kolmogorov–Smirnov test and statistical tests were selected based on these results. All data values in the text and figures are presented as mean ± SEM.

## Results

### MSN subtypes and their projections in BAC *drd1a*-tdTomato;BAC *drd2*-EGFP mice through development

**Figure 1:**
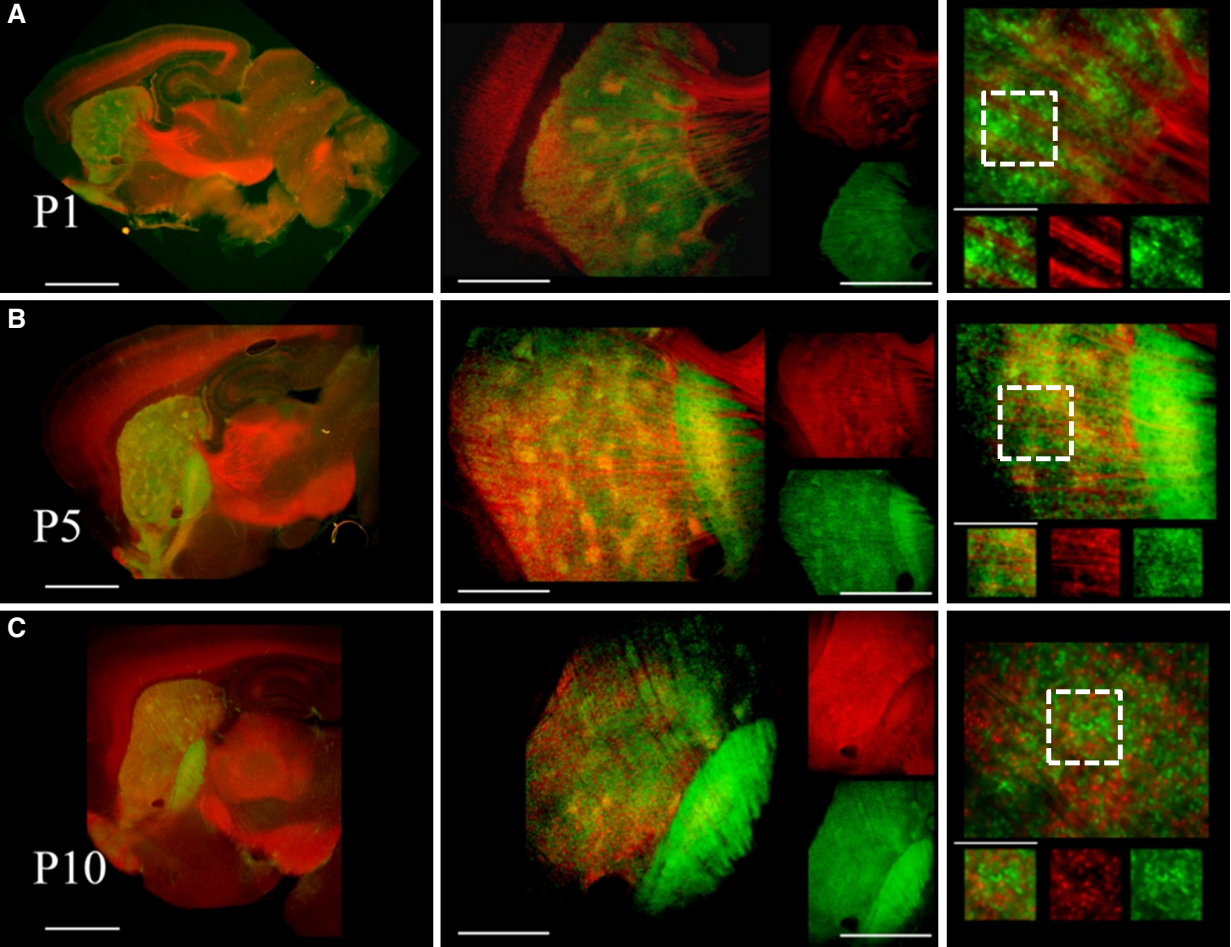
Distribution of striatonigral and striatopallidal neurons and their projections in BAC *drd1a*-tdTomato;BAC *drd2*-EGFP mice through early development. Photomicrographs of fixed sagittal slices (150 *μ*m) from mice at P1 (A), P5 (B), and P10 (C). Slices in the left column are further magnified in the middle and right columns. The inset in the white dashed boxes are magnified below (merged on left, channels separated in middle and right), illustrating a lack of colocalization between red and green cells through early development. Calibration bars are from left to right: 0.5 mm, 0.25 mm 0.45 mm, and 0.15 mm.

To study dopamine receptor expression through development, we compared fluorophore expression in fixed sagittal slices of BAC *drd1a*-tdTomato;BAC *drd2*-EGFP mice at postnatal days 1 (P1), 5, and 10 (Fig. 1). Both fluorophores were visible at P1 suggesting the presence of D1 and D2 receptors. However, compared to the relatively stable striatal D2 receptor expression by P1, striatal D1 receptor expression appeared to increase from P1 to P10. This may be due to the low the signal-to-noise ratio of tdTomato in these mice at early time points, as recently suggested in Thibault et al. (2013).

D1 receptor containing MSNs (D1 MSNs) project directly to the substantia nigra pars reticulata (SN_pr_), and D2 receptor containing MSNs (D2 MSNs) project indirectly to the SN_pr_ via the external segment of the globus pallidus (Gong et al. 2003; Shuen et al. 2008). In BAC *drd1a*-tdTomato;BAC *drd2*-EGFP mice, D1 MSN fibers from the striatum visibly projected to the substantia nigra by P1 (Fig. 1A), and by P5 D2 MSN fibers projected to their target nucleus, the external globus pallidus (Fig. 1B). In line with previous findings of D1 receptor expressing neurons in the cortex (Gong et al. 2003), we also observed consistent tdTomato expression in the cortex through development.

### MSNs in coculture develop considerably between first and second week in vitro

**Figure 2:**
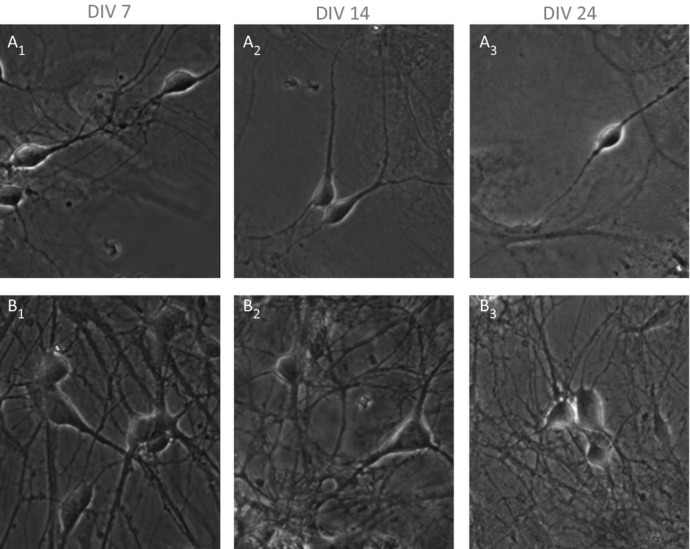
Phase contrast micrographs of DIV 7 (A_1_, B_1_), 14 (A_2_, B_2_), and 24 (A_3_, B_3_) neurons plated without (A_1–3_) and with (B_1–3_) BDNF and GDNF, illustrating that corticostriatal cultures plated in the presence of 50 ng/mL BDNF and 30 ng/mL GDNF develop more diverse phenotypes and retain greater neuron density.

Brains were harvested for cultures at P0-1. As D1 and D2 receptors are expressed in the cortex by P1 (Fig. 1A), cortices were selected from littermates that expressed neither fluorophore. Mice that expressed both fluorophores were used for their striatal tissues, and exact dissection was possible due to the strong expression of D2 receptor in the striatum (Lalchandani et al. 2013). The inclusion of 50 ng/mL BDNF and 30 ng/mL GDNF in the feeding media as previously reported in Gertler et al. (2008) and Tian et al. (2010) drastically increased the diversity and complexity of the cell types observed in the cultures (Fig. 2). Density of MSNs with fluorophore expression did not significantly change between the first and second week in vitro: D1 MSN density decreased from 66 ± 10 to 48 ± 6 cells/mm^2^ and D2 MSN density increased from 31 ± 15 to 43 ± 5 cells/mm^2^, respectively. Colocalization of both fluorophores was detected in 5–10% of the neurons and their density also remained stable through development: 12 ± 5 cells/mm^2^ and 14 ± 3 cells/mm^2^ overlapped at the first and second week in vitro, respectively (34 cells from four distinct culture preparations). Previous studies of dopamine receptor expression in pre- or early postnatal rodents suggest that the expression of D1 and D2 receptors colocalizes to a greater degree at earlier time points (Goffin et al. 2010; Thibault et al. 2013). While these neurons may be of great interest in striatal development, they were avoided in the current study.

**Figure 3:**
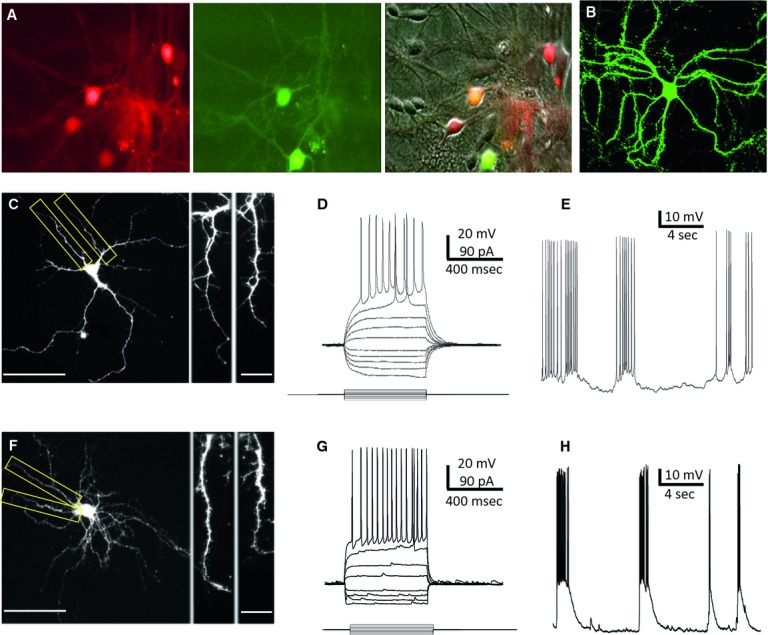
Primary corticostriatal cocultures from BAC *drd1a*-tdTomato;BAC *drd2*-EGFP mice. (A) Examples of cocultures of cortical and striatal neurons in vitro at DIV 15. Red neurons (*left*) are D1 MSNs, green neurons (*middle*) are D2 MSNs. The two images are also shown (*right*) superimposed to the DIC image. Yellow neurons indicate colocalization. (B) Confocal laser scanning microscopy image of a D2 MSN from *Drd1a*-tdTomato/*Drd2*-EGFP culture at DIV 21. GFP signal is amplified with *α*-GFP. Images of a D2 neuron at DIV 7 (C) and DIV 14 (F). Insets of dendritic segments are shown on the right and illustrate the typical conversion from filopodia to spines with development (calibration bars are 100 *μ*m left, 25 *μ*m right). Voltage traces from patch-clamp recordings from cultured MSNs at DIV 7 (D) and DIV 14 (G) with K-gluconate-based intracellular solution (IS_1_) illustrate responses to increasing current injections. Spontaneous membrane potential fluctuations illustrate the occurrence of putative “up” and “down states” in MSNs recorded in current clamp at DIV 7 (E) and DIV 15 (H).

Neurons in culture were easily identifiable with a light microscope equipped with phase contrast and fluorescence, and fluorophore expression allowed for targeted recordings from distinct MSNs (Fig. 3A). To study membrane properties of neurons in culture at early and late time points, whole-cell patch-clamp recordings were conducted from MSNs during the first (DIV 7–10) and second (DIV 13–16) week in vitro. While many properties were comparable between MSN subtypes, there was an effect of time in vitro on RMP in D1 MSNs, capacitance in D2 MSNs, and input resistance in both subtypes (Table 1).

**Table 1:** Membrane properties of D1 and D2 MSNs in primary cultures through development

	DIV 7–10		DIV 13–16	
		
	D1+	D2+	D1+	D2+
RMP (mV)	−52 ± 14 (*n* = 5)	−58.3 ± 2.9 (*n* = 11)	−62.3 ± 2.2 (*n* = 17)	−64.6 ± 2.3 (*n* = 31)
Capacitance (pF)	32 ± 8 (*n* = 5)	35 ± 5 (*n* = 7)	51 ± 13 (*n* = 4)	50 ± 4+ (*n* = 16)
Input resistance (mΩ)	822 ± 201 (*n* = 14)	891 ± 331 (*n* = 12)	362 ± 46 (*n* = 15)	314 ± 39* (*n* = 28)
Inward rectification index	1.1 ± 0.1 (*n* = 3)	1.3 ± 0.5 (*n* = 4)	2.2 ± 0.7 (*n* = 4)	2.2 ± 0.3 (*n* = 18)

Whole-cell recordings from identified MSNs suggest that, while there are changes observed between one (DIV 7–10) and two (DIV 13–16) weeks in vitro, there is no difference between the D1 and D2 MSNs at either time point. Resting membrane potential (RMP) values are not corrected for liquid junction potential. Comparing MSN subtypes between the first and second week in vitro:

+ *P* < 0.05 using an unpaired t-test for normally distributed data, and

* *P* < 0.05 using a Mann–Whitney test for data that was not normally distributed.

Time in vitro also altered MSN morphology: MSNs at DIV 14 displayed more extensive dendritic complexity and more mature spines in comparison to DIV 7 (Fig. 3C and F). Active membrane properties became more mature between the first and second week, as seen with differences in action potential firing patterns (Fig. 3D and G) and up-and-down states (Fig. 3E and H) (Randall et al. 2011).

### Cocultures express functional GABA_A_ receptors

**Figure 4:**
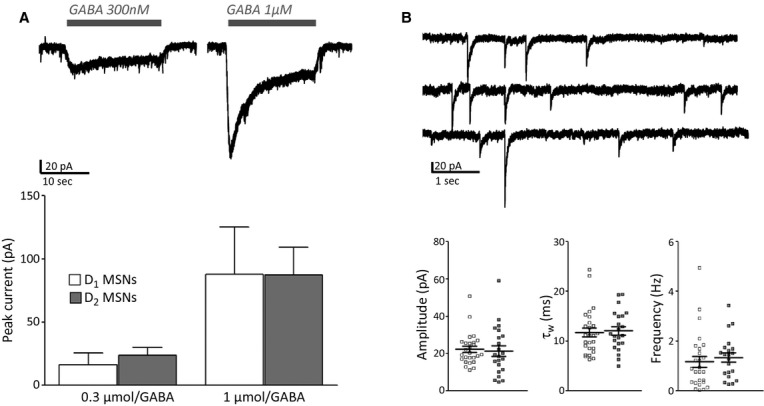
Cocultures express functional GABA_A_ receptors. (A) Whole-cell currents in a representative D2 MSN demonstrate responses to two GABA concentrations at DIV 15 (*top*). Summary plots of peak responses to GABA illustrate the presence of GABA_A_ receptors on both MSN subtypes at DIV 14–16 (*bottom*). (B) mIPSCs recorded in a D2 MSN at DIV 15 (*top*). Summary scatter plots show no differences in mIPSC amplitude, decay, and frequency (*bottom*; *n* = 26 D1 MSNs, *n* = 22 D2 MSNs). A KCl-based intracellular solution, IS_2_, was used for both experiments.

We next assessed sensitivity to GABA by locally applying GABA in cultures at 2 weeks in vitro. As cells were dialyzed with a high concentration of chloride (IS_2_, *Methods*), GABA_A_ receptor opening resulted in inward currents. Twenty-second applications of GABA at three distinct doses revealed two phases of response—a peak and a plateau—to low GABA concentrations (Fig. 4A, top). Both D1 and D2 MSNs displayed similar sensitivities to GABA (Fig. 4A, bottom).

As the GABA_A_ receptor is a pentameric structure with different subunits conferring different channel properties, subunit selective pharmacology was also applied to dissect out potential differences between MSN subtypes in these culture preparations (Pirker et al. 2000). To target the extrasynaptic *δ* subunit containing GABA_A_ receptor, we locally applied the *δ* preferring agonist THIP (Brown et al. 2002). Interestingly, currents in response to THIP were larger in D2 MSNs, suggesting a higher expression of the *δ* subunit. In addition, we tested the GABA_A_ receptor *β*2/3 subunit selective agonist etomidate on whole-cell currents, which again revealed an increase in *β*2/3 expression in D2 MSNs in vitro. These data are summarized in Table 2.

**Table 2:** GABA currents in D1 and D2 MSNs in primary cultures at DIV 14–16

	0.3 *μ*mol/L GABA	1 *μ*mol/L GABA	3 *μ*mol/L GABA	1 *μ*mol/L THIP	5 *μ*mol/L Etom
Peak (pA)					
D1	20 ± 12 (*n* = 13)	97 ± 42 (*n* = 14)	228 ± 72 (*n* = 8)	7 ± 2 (*n* = 14)	90 ± 26 (*n* = 15)
D2	24 ± 7 (*n* = 20)	88 ± 22 (*n* = 21)	477 ± 96 (*n* = 10)	18 ± 3** (*n* = 19)	136 ± 19* (*n* = 21)
Steady state (pA)					
D1	13 ± 6 (*n* = 13)	33 ± 13 (*n* = 14)	86 ± 24 (*n* = 8)	4 ± 1 (*n* = 14)	55 ± 12 (*n* = 15)
D2	16 ± 4 (*n* = 20)	39 ± 9 (*n* = 21)	142 ± 35 (*n* = 10)	14 ± 2*** (*n* = 19)	81 ± 10 (*n* = 21)

Local application of three concentrations of GABA, of *δ* subunit preferring agonist THIP and of *β*2/3 selective agonist etomidate on whole-cell currents of identified MSNs. The peak current values are the maximum amplitude of the response, and steady-state values are measured at 6–8 sec after application. *P < 0.05 **P < 0.01 and ***P < 0.001, comparing MSN subtypes using a Mann–Whitney test.

To study single GABAergic synapses of MSNs in vitro, we isolated miniature inhibitory postsynaptic currents (mIPSCs) with local application of 5 μmol/L NBQX and 0.5 μmol/L TTX. Although mIPSCs may be derived from presynaptic terminals from cortical GABAergic neurons, striatal interneurons or MSNs, the occurrence of mIPSCs indicates the successful innervation and presence of postsynaptic GABA_A_ receptors. mIPSCs were studied in both D1 and D2 MSNs at 2 weeks in vitro, and the kinetics and frequency of these events were comparable across the two subtypes of MSNs (Fig. 4B).

### Ease in study of MSN inhibitory collaterals

**Figure 5:**
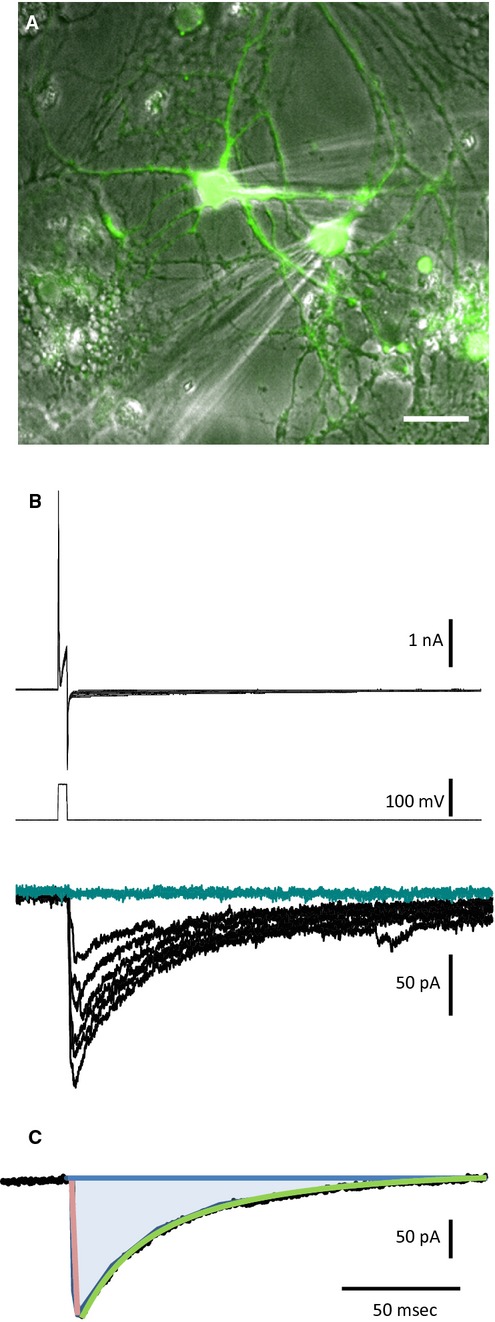
Paired recordings reveal large evoked IPSCs between MSN subtypes. (A) Superimposed eGFP fluorescence and DIC images illustrating an example of a dual patch-clamp recording from a pair of D2 MSNs (calibration bar, 25 *μ*m). (B) Example traces of evoked synaptic currents in a D2 neuron at DIV 15. A 100 mV test pulse is given to the presynaptic neuron (*top*), creating an action current (*middle*) and an evoked current in the postsynaptic neuron (*bottom*), showing amplitude fluctuation. Local application of BMR (*blue*) eliminates evoked currents. (C) Inhibitory collaterals can be studied by averaging the eIPSC to look at amplitude and rise time (*pink*), decay (*green*), and charge transfer (*blue*) of the eIPSC. eIPSCs were studied with IS_2_.

Previous studies have detected an increased rate of coupling between neurons in vitro when compared to ex vivo slice (Czubayko and Plenz 2002; Tunstall et al. 2002; Plenz 2003). Neuron collaterals can be studied by performing paired patch-clamp recordings and eliciting an action current with a single depolarizing voltage step (Fig. 5A and B). Here, we increased the internal chloride concentration in the recording pipette to increase detection of inhibitory currents (IS_2_, *Methods*). eIPSCs were detected in 13% of pairs at DIV 7–8 (*n* = 8) and 42% of recorded pairs at DIV 13–16 (*n* = 96) in five culture preparations. eIPSCs detected at 1 week in vitro were exclusively between D1 MSNs. Autaptic eIPSCs were also often detected in voltage clamp, where the elicited action potential revealed an IPSC in the same neuron. These autaptic currents were found in 50% of MSNs at DIV 7–8 (*n* = 8) and 57% of MSNs at DIV 13–16 (*n* = 96). Local application of BMR eliminated synaptic eIPSCs, autaptic eIPSCs, and most spontaneous activity, indicating that the synapses between MSNs in vitro are GABAergic collaterals (Fig. 5B, blue traces). While BMR has been shown to have off-target effects on small conductance calcium-activated potassium channels (Debarbieux et al. 1998), the water soluble and quickly reversible nature of BMR application outweighed the potential non-GABAergic effect.

In the first week in vitro, only eIPSCs between D1 MSNs were detected, although sIPSCs occurred at low frequency in both D1 and D2 MSNs. By the second week in vitro, synapses had formed between all possible MSN pair combinations (Table 3). Directly stimulating targeted neurons typically resulted in a fast latency to peak (5.2 ± 0.5 msec, *n* = 33; 4 culture preparations) and single-peaked responses, indicating that eIPSCs are the result of the activation of a specific presynaptic MSN rather than of polysynaptic activity. Taking advantage of this configuration, we studied the properties of these identified collaterals (Fig. 5C, Table 3). As the rate of synapse formation at the first week in vitro was low, this analysis was limited to DIV 14–16.

**Table 3:** Properties of evoked IPSCs in D1 and D2 MSNs in primary cultures at DIV 13–16

	D1→D1	D1→D2	D2→D1	D2→D2
% Coupled	26% (*n* = 42)	47% (*n* = 15)	40% (*n* = 15)	67% (*n* = 24)**
Peak (pA)	174 ± 70 (*n* = 12)	58 ± 11 (*n* = 9)	100 ± 18 (*n* = 6)	126 ± 38 (*n* = 11)
Tw (msec)	72 ± 8 (*n* = 12)	75 ± 9 (*n* = 9)	59 ± 11 (*n* = 6)	81 ± 21 (*n* = 11)
Charge (nA msec)	10 ± 4 (*n* = 12)	4 ± 1 (*n* = 9)	6 ± 1 (*n* = 6)	7 ± 3 (*n* = 11)
CV	0.30 ± 0.04 (*n* = 12)	0.30 ± 0.03 (*n* = 9)	0.46 ± 0.06 (*n* = 6)	0.40 ± 0.05 (*n* = 11)
% Failures	10 ± 6 (*n* = 12)	5 ± 4 (*n* = 9)	4 ± 4 (*n* = 6)	4 ± 1 (*n* = 11)
Quantal content	18 ± 15 (*n* = 12)	3 ± 0.3 (*n* = 9)	5 ± 1.5 (*n* = 6)	16 ± 9 (*n* = 11)

Four combinations of MSN pair recordings were conducted and eIPSCs were elicited in voltage clamp (see *Methods*). In the first row, **P < 0.01, comparing D1–D1 pairs to D2–D2 pairs using a *χ*2 test. In all subsequent rows, statistics were conducted using Kruskal–Wallis test with Dunns posttest to compare MSN pairs.

**Figure 6:**
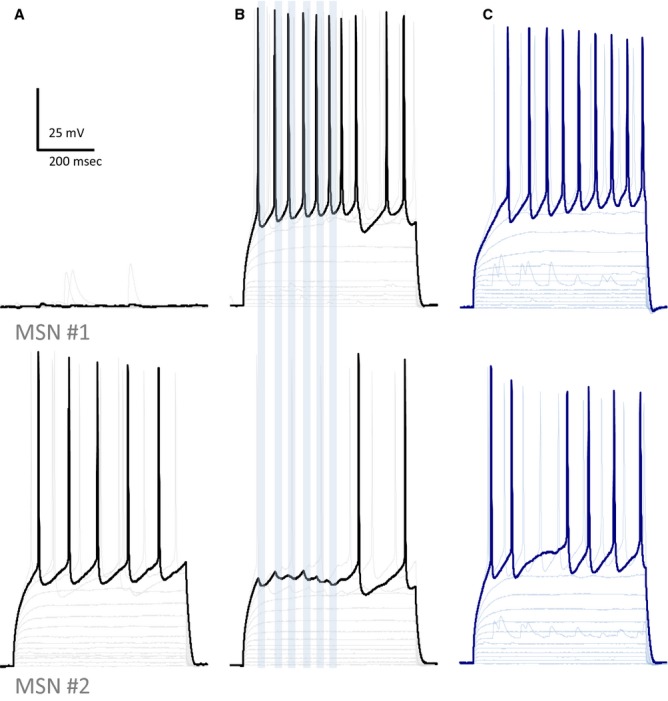
Lateral inhibition shapes firing patterns. Paired recording from two DIV 27 D2 MSNs with IS_1_: MSN#1 (*top*) is unidirectionally coupled to MSN#2 (*bottom*). In panel (A) MSN #1 is at rest, while MSN#2 is injected with increasing 10 pA current steps, eliciting action potential firing. (B) When MSN #1 is also induced to fire action potentials, the firing rate in MSN#2 is attenuated despite receiving the same current steps as in panel A. Note that IPSPs are apparent in MSN#2 (blue bars). (C) In the presence of 25 *μ*mol/L BMR both MSNs fire at a lower rate than what is observed in panel (B) and IPSPs are eliminated. Traces in bold are voltage responses to a 250 pA current injection, and all panels receive a maximum of 270 pA.

These high-frequency MSN collaterals have the ability to shape the firing patterns of neurons they are coupled with and are therefore important in striatal circuit output. Figure 6 is an example of two MSNs that are unidirectionally coupled and in current clamp configuration. MSN#1 is coupled to MSN#2, as determined by the stimulation protocol described in Figure 5. While MSN#1 is at rest, MSN#2 fires action potentials in response to increasing current injections (Fig. 6A). When MSN#1 is also induced to fire, the firing rate in response to the same number of current steps in MSN#2 is considerably attenuated (Fig. 6B). Upon closer examination, with every action potential in MSN#1, there is an inhibitory postsynaptic potential (IPSP) in MSN#2 (Fig. 6B, blue bars). If BMR is locally applied while both MSN #1 and #2 are induced to fire, these IPSPs disappear, demonstrating GABAergic coupling between MSNs. In addition, BMR application reduces the firing rate in both cells, despite following the same current step protocol as in Figure 6B, similar to what previously reported in Ade et al. (2008).

## Discussion

The striatum holds a critical role in information processing within the basal ganglia and is therefore a subject of high interest for the understanding of movement control and their pathologies. Neuron collaterals endow the striatum with the ability to process and consolidate multiple inputs. This information is relayed to the SN_pr_, resulting in executive action. Improper striatal function results in a slew of pathological states including Parkinson's disease, and striatal collaterals have been largely implicated with changes in dopamine levels (Taverna et al. 2008; Lalchandani et al. 2013). Given the infrequent detection of MSN collaterals in the traditional slice preparation, here we have developed a dissociated culture model, in which collaterals are more frequent, and in which MSNs are identifiable by fluorophore expression.

Previous studies have reported a range of connectivity rates between D1 and D2 MSNs, with variations in methodology potentially leading to diverse results (Jaeger and Wilson 1994; Czubayko and Plenz 2002; Tunstall et al. 2002; Koos et al. 2004; Venance et al. 2004; Gustafson et al. 2006; Tecuapetla et al. 2007; Taverna et al. 2008). In these studies, functional coupling was reported at higher rates in culture models compared to ex vivo slices. Here, we report the same trend: at 2 weeks in vitro, we detect connections between 41.7% MNSs. And although dissociated culture models have inherent limitations, such as removing structures from their native environment and bathing neurons in predetermined concentrations of growth factors, conventional ex vivo slices also come with certain constraints. Given that MSN arbors extend beyond the typical 250–400 *μ*m slice preparation (Wilson 1994), differences in slice thickness and depth of neurons selected can easily affect the percentage of coupling detected. And while dissociated primary cultures are grown in an artificial environment, neurites are not severed after plating, allowing for the study of intact neuron collaterals. Ultimately, it is important to keep in mind the limitations of each preparation when interpreting results.

Primary culturing techniques have been utilized for many years and recent advances suggest eliminating cytosine arabinofuranoside (Martin et al. 1990), reducing or removing serum (Ahmed et al. 1983), and including low concentrations of growth factors (Gertler et al. 2008). Our protocol has amalgamated these past advances, further demonstrating that the presence of growth factors plays a fundamental role in proper neuronal maturation. Neurons in these cultures retained the native expression of fluorophores and MSNs had passive and active membrane properties that were reminiscent of those observed in ex vivo slice recordings.

Neurons in our cultures displayed substantial development between the first and second week in vitro. By DIV 13, MSNs exhibited reduced input resistance and more hyperpolarized membrane potentials, suggesting an increased expression and opening of inward-rectifier potassium channels with time (Jiang and North 1991). Additionally, the rates of connectivity observed between MSNs at the first week in vitro were less than 15% and restricted to only D1 MSNs collaterals. The delayed development of D2 MSN collaterals may be correlated with the findings in Figure 1, which suggest that D2 MSN projections to the globus pallidus take longer to develop than D1 MSN projections to the SN in sagittal brain slices. MSNs in the cultures also expressed GABA_A_ receptors, as determined by whole-cell currents in response to low doses of GABA. Although there was no difference in GABA sensitivity between MSN subtypes, there appeared to be differential subunit expression: D2 MSNs displayed larger responses to *β*2/3 and *δ* subunit-specific agonists. The larger *β*2/3-subunit-mediated currents support data previously collected in our laboratory from ex vivo mice striatal slices, suggesting that D2 MSNs have a higher expression of *β*2/3 subunits (Ade et al. 2008; Janssen et al. 2009). However, the larger currents in response to the *δ* subunit-specific agonist THIP is in contrast to what our laboratory has reported for D2 MSNs in young mouse brain slices (Ade et al. 2008). This may be indicative of specific culturing conditions causing upregulation of receptor subtypes responsible for *δ*-subunit-mediated tonic currents, or of an accelerated rate of maturity in vitro, as mature mouse slices have been reported to have larger *δ*-subunit-mediated tonic currents (Santhakumar et al. 2010).

Neural circuits are defined by their connections. Consequently, MSN collaterals, which have the capacity to simultaneously integrate multiple inputs, have the ability to effectively regulate striatal output. Timing of MSN collateral activation further defines the output to the SN_pr_, as MSN collaterals can be either depolarizing or hyperpolarizing dependent upon the membrane potential of the receiving MSN. Given that long-term potentiation and depression are reliant on both glutamatergic input onto MSNs and spike timing of the MSNs themselves (Shen et al. 2008), MSN collaterals are at the fulcrum of striatal activity and should be studied in further detail.

## Conclusions

D1 and D2 MSNs extend inhibitory collaterals that shape neuron firing and striatal output. Using BAC transgenics and paired whole-cell recordings, we describe a paradigm that allows for the simultaneous identification of both MSN subtypes and the targeted study of MSN collaterals.
